# Short-term and long-term efficacy in robot-assisted treatment for mid and low rectal cancer: a systematic review and meta-analysis

**DOI:** 10.1007/s00384-023-04579-3

**Published:** 2023-12-21

**Authors:** Huiming Wu, Renkai Guo, Huiyu Li

**Affiliations:** Department of General Surgery, Third Hospital of Shanxi Medical University, Shanxi Bethune Hospital, Shanxi Academy of Medical Sciences Tongji Shanxi Hospital, Taiyuan, 030032 China

**Keywords:** Robot-assisted, Mid and low rectal cancer, Short-term efficacy, Long-term efficacy, Meta-analysis

## Abstract

**Objective:**

This study aims to conduct a meta-analysis to evaluate the short-term and long-term therapeutic effects of robot-assisted laparoscopic treatment in patients with mid and low rectal cancer.

**Methods:**

A comprehensive search strategy was employed to retrieve relevant literature from PubMed, NCBI, Medline, and Springer databases, spanning the database inception until August 2023. The focus of this systematic review was on controlled studies that compared the treatment outcomes of robot-assisted (Rob) and conventional laparoscopy (Lap) in the context of mid and low rectal cancer. Data extraction and literature review were meticulously conducted by two independent researchers (HMW and RKG). The synthesized data underwent rigorous analysis utilizing RevMan 5.4 software, adhering to established methodological standards in systematic reviews. The primary outcomes encompass perioperative outcomes and oncological outcomes. Secondary outcomes include long-term outcomes.

**Result:**

A total of 11 studies involving 2239 patients with mid and low rectal cancer were included (3 RCTs and 8 NRCTs); the Rob group consisted of 1111 cases, while the Lap group included 1128 cases. The Rob group exhibited less intraoperative bleeding (MD =  −40.01, 95% CI: −57.61 to −22.42, *P* < 0.00001), a lower conversion rate to open surgery (OR = 0.27, 95% CI: 0.09 to 0.82, *P* = 0.02), a higher number of harvested lymph nodes (MD = 1.97, 95% CI: 0.77 to 3.18, *P* = 0.001), and a lower CRM positive rate (OR = 0.46, 95% CI: 0.23 to 0.95, *P* = 0.04). Additionally, the Rob group had lower postoperative morbidity rate (OR = 0.66, 95% CI: 0.53 to 0.82, *P* < 0.0001) and a lower occurrence rate of complications with Clavien–Dindo grade ≥ 3 (OR = 0.60, 95% CI: 0.39 to 0.90, *P* = 0.02). Further subgroup analysis revealed a lower anastomotic leakage rate (OR = 0.66, 95% CI: 0.45 to 0.97, *P* = 0.04). No significant differences were observed between the two groups in the analysis of operation time (*P* = 0.42), occurrence rates of protective stoma (*P* = 0.81), PRM (*P* = 0.92), and DRM (*P* = 0.23), time to flatus (*P* = 0.18), time to liquid diet (*P* = 0.65), total hospital stay (*P* = 0.35), 3-year overall survival rate (*P* = 0.67), and 3-year disease-free survival rate (*P* = 0.42).

**Conclusion:**

Robot-assisted laparoscopic treatment for mid and low rectal cancer yields favorable outcomes, demonstrating both efficacy and safety. In comparison to conventional laparoscopy, patients experience reduced intraoperative bleeding and a lower incidence of complications. Notably, the method achieves comparable short-term and long-term treatment results to those of conventional laparoscopic surgery, thus justifying its consideration for widespread clinical application.

**Supplementary Information:**

The online version contains supplementary material available at 10.1007/s00384-023-04579-3.

## Background

Laparoscopic minimally invasive surgery has become widely accepted for the treatment of colorectal cancer globally [1]. However, when compared to colon cancer, surgical procedures for mid and low rectal cancer are inherently more intricate, presenting specific surgical demands and challenges within the operative environment. Total mesorectal excision (TME) stands as the gold standard for curative surgery in rectal cancer, ensuring the complete removal of the rectum and surrounding lymph nodes to achieve a circumferentially negative surgical margin [2]. Modern surgical approaches also emphasize the preservation of pelvic autonomic nerve function to enhance patients’ quality of life, requiring advanced surgical skills [3]. The complex structure of pelvic organs, distributed within a confined space containing numerous critical organs, vessels, and nerves, makes it challenging for laparoscopic instruments with elongated handles to navigate, thereby increasing the difficulty of the surgical procedure [4]. In recent years, the application of the da Vinci robotic surgical system (Intuitive Surgical Inc., Sunnyvale, CA) in rectal cancer has seen gradual growth. The advantages of the robotic system, such as a magnified 3D field of view, 10 times enlargement, and 7 degrees of freedom in wrist-like surgical instruments, have successfully overcome limitations in the laparoscopic approach, particularly in terms of surgical visibility and precise manipulation [2]. These features make the robotic system particularly suitable for performing surgery in the confined space of the pelvic cavity. While robotic surgery appears to be a safe and feasible surgical approach in colorectal cancer, however, there is still some controversy in clinical application. Therefore, we conducted a recent meta-analysis study, comparing the short-term and long-term treatment outcomes of robotic and laparoscopic surgery for mid and low rectal cancer. This research aims to provide a more comprehensive understanding of the relative effectiveness of these two surgical approaches, offering clearer guidance for treatment decisions in patients with rectal cancer.

## Methods

### Study design

The protocol was compiled and registered in PROSPERO (CRD42023466246). We conducted this meta-analysis in accordance with the Preferred Reporting Items for Systematic Reviews and Meta-analysis: the PRISMA statement [5]. Methodological quality was ensured by following AMSTAR (assessing the methodological quality of systematic review) guidelines.

A systematic search for published articles was conducted in August 2023 in four electronic databases (PubMed, NCBI, Medline, and Springer databases,) from their inception till the end of July 2023. The keywords and Medical Subject Headings terms used for the search strategy were rectal neoplasm OR rectal cancer OR rectal carcinoma OR rectal tumor AND robotics OR robotic surgery OR robot assisted laparoscopy OR robot assisted total mesorectal excision AND laparoscopy OR laparoscopic surgery OR laparoscopic total mesorectal excision OR laparoscopic TME AND randomized controlled trial OR Retrospective study. References of accepted articles were also manually screened for potentially relevant studies to ensure that no additional publications were missed.

The selection criteria followed the PICOT. P (participants): patients over 18 years of age diagnosed with mid or low rectal cancer; I (intervention): robot-assisted laparoscopic resection; C (comparison): laparoscopic rectal resection; O (outcomes): short-term and long-term therapeutic effects; T (type of study design): randomized controlled trial and retrospective study.

The last search was performed in the end of August 2023, the search strategy was limited to papers written in English, and the reference lists of the eligible studies were tracked manually for other potentially relevant studies.

### Eligibility criteria and study selection

Two independent authors (HMW and RKG) meticulously sifted through articles retrieved from the initial literature search, meticulously removing duplicate studies and excluding those not directly pertinent to the research. The two authors then independently conducted a detailed review of studies that met the predetermined eligibility criteria, whether in abstract or full-text form, carefully evaluating their alignment with the specified standards. Any disparities in study selection were methodically addressed through thorough discussions, consensus-building, or seeking input from a third independent author (HYL). The inclusion criteria were thoughtfully outlined as follows: (1) the study designs were exclusively centered on comparing robotic-assisted and conventional laparoscopic treatments for rectal cancer; (2) each group consisted of a minimum of 10 patients; (3) all subjects were clinically and pathologically confirmed or diagnosed through laboratory examinations as having mid or low rectal (tumor located within 15 cm of the anus) cancer; (4) crucial data required for this study could be reliably obtained, and statistical methods were diligently applied; and (5) the literature had undergone prior public dissemination.

### Data extraction

Data extraction from the enrolled studies was undertaken independently by two meticulous authors, HMW and RKG. Any disparities in the extraction process were diligently addressed through comprehensive discussions and, when needed, consultation with the third author (HYL):

1. Characteristics of included studies

Author’s name; year of publication; study design; sample size, age, sex; body mass index (BMI); American Society of Anesthesiologists (ASA) grading; cTMN stage; tumor size; tumor distance from the anal verge (Endoscopic diagnosis); neoadjuvant therapies; and follow-up duration.

2. Primary outcomes

Conversion to open surgery rate, total hospital stay, postoperative complications, circumferential resection margin positive rate.

3. Secondary outcomes

Operation time, operative blood loss, protective stoma rate, time to flatus, time to liquid diet, occurrence rate of complications with Clavien–Dindo grade ≥ 3 [6], harvested lymph nodes, proximal resection margin, distal resection margin, 3-year overall survival rate, 3-year disease-free survival rate.

### Risk of bias assessment

The quality of the RCTs included in the analysis was assessed using the Cochrane Collaboration’s tool [7] for evaluating the risk of bias, while NRCTs were evaluated using the Risk of Bias in Non-Randomized Studies of Interventions (ROBINS-I) tool.

### Statistical analysis

Data organization and calculations were conducted using Excel 2016 software, and data analysis was performed using RevMan 5.4 software. For continuous variables, mean difference (MD) was employed. Given variations in assessment methods across included studies, standardized mean difference (SMD) was used as the composite effect measure to eliminate the impact of differing data scales. Categorical variables were addressed using odds ratio (OR). Heterogeneity analysis employed the *χ*^2^ test [8]. If *P* ≤ 0.1 and *I*^2^ ≥ 50%, it indicated significant heterogeneity among included studies, prompting the adoption of a random effects model (REM); otherwise, a fixed effects model (FEM) was chosen. A significance level of *P* ≤ 0.05 was considered statistically meaningful. A funnel plot was created to assess publication bias.

### Trial sequential analysis (TSA)

To mitigate the risk of type I errors associated with repeated significance testing and minimize the potential for false positive results due to random errors, we employed trial sequential analysis (TSA) 0.9.5.10 Beta software to conduct sequential analyses on the advantageous outcomes identified in the robotic (Rob) group during this analysis process.

## Result

### Literature searching

**Fig. 1:**
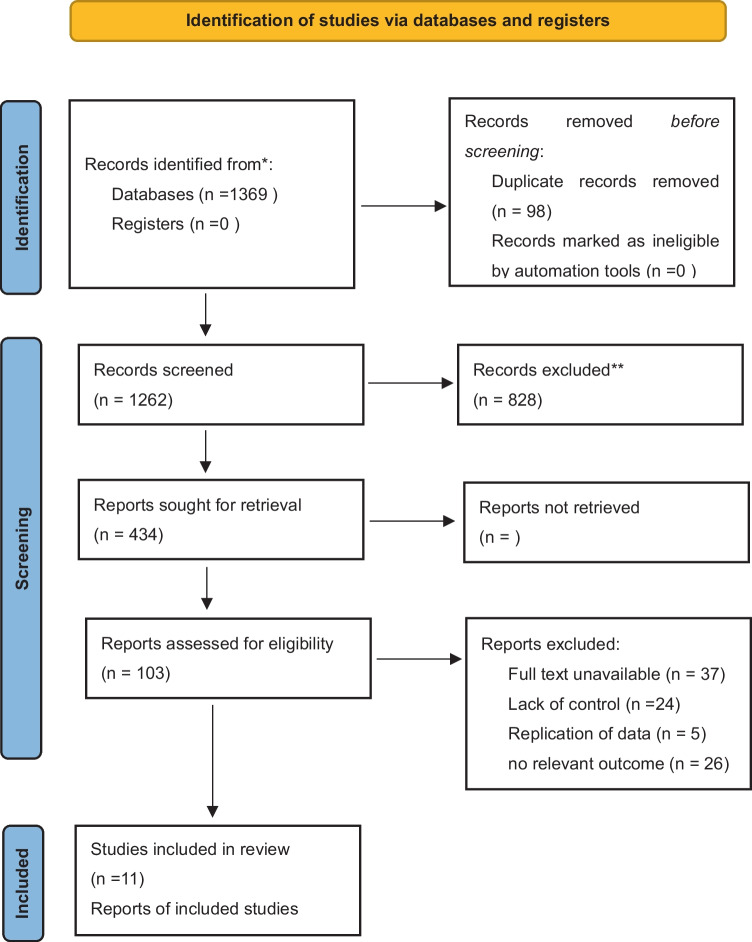
PRISMA flow chart of study selection. *Consider, if feasible to do so, reporting the number of records identified from each database or register searched (rather than the total number across all databases/registers). **If automation tools were used, indicate how many records were excluded by a human and how many were excluded by automation tools. *From:* Page MJ, McKenzie JE, Bossuyt PM, Boutron I, Hoffmann TC, Mulrow CD, et al. The PRISMA 2020 statement: an updated guideline for reporting systematic reviews. BMJ 2021;372:n71. 10.1136/bmj.n71

During the initial screening, a total of 1389 studies were identified. After removing duplicates, we screened 1262 studies and identified 103 eligible studies by reviewing titles and abstracts. Among these 103 studies, 11 articles were determined to meet the inclusion criteria for the final analysis after a full-text evaluation. The study selection progress is presented in the PRISMA flow diagram (Fig. 1).

### Characteristics of the included studies

A total of 11 studies, involving 2239 patients with mid and low rectal cancer, were included—comprising 3 RCTs and 8 NRCTs. The robotic group consisted of 1111 cases, while the Lap group had 1128 cases. The mean age ranged from 53.1 to 66, and the male-to-female ratio was 1.7:1. The mean BMI varied from 21.9 to 26 kg/m^2^. Patients with ASA I scores accounted for 25.2 to 67.1%, ASA II accounted for 29 to 57.7%, ASA III accounted for 0 to 33.3%, and ASA IV accounted for 0 to 0%. Patients with TMN stage I accounted for 0 to 55.0%, TMN stage II accounted for 17.1 to 46.5%, TMN stage III accounted for 11 to 57.1%, and TMN stage IV accounted for 0 to 7.9%. The mean tumor size ranged from 2.5 to 3.6 cm. The mean distance from the anal verge ranged from 3.24 to 8 cm. Six studies described the follow-up duration. The characteristics of included studies are summarized in Table 1.

**Table 1:** Characteristics of the included studies

Author	Year	Study design	Group	Patients	Mean age	Sex (M/F)	Mean BMI	ASA (I/II/III/IV)	TMN stage (I/II/III/IV)	Tumor size (cm)	Distance from the anal verge (cm)	Neoadjuvant therapies	Follow-up duration (months)
Bedirli et al. [9]	2016	NRCT	Rob	35	64.7 ± 8.5	24/11	24.7 ± 3.9	6/17/12/0	0/13/19/3	———	———	35	——
			Lap	28	60.4 ± 7.1	19/9	23.2 ± 3.2	4/15/9/0	0/9/17/2	———	———	28	——
Baek et al. [10]	2013	NRCT	Rob	47	58.0 ± 12.9	31/16	23.37 ± 3.27	22/24/1/0	28/9/10/1	———	4.39 ± 2.25	——	60
			Lap	37	61.8 ± 12.8	28/9	23.4 ± 2.74	25/12/0/0	17/9/8/3	———	5.52 ± 3.74	——	60
Feng et al. [11]	2021	NRCT	Rob	137	58.3 ± 11.2	75/62	——	93/38/6/0	54/24/59/0	3.3 ± 1.1	6.7 ± 2.0	5	72
			Lap	137	59.5 ± 11.2	81/56	——	91/41/5/0	54/22/61/0	3.4 ± 1.0	6.9 ± 1.8	7	72
Feng et al. [12]	2022	RCT	Rob	586	59·1 ± 11·0	356/230	23.5 ± 3.3	324/230/32/0	205/192/189/0	——	5.9 ± 2.4	254	——
			Lap	585	60.7 ± 9.8	354/231	23.5 ± 3.1	318/240/27/0	203/200/182/0	——	5.8 ± 2.6	257	——
Feroci et al. [13]	2016	NRCT	Rob	53	66 (33–80)	42/16	24.6 (19–37)	11/33/9/0	27/8/18/0	——	8 (3–12)	26	80
			Lap	58	66 (42–84)	27/26	24.6 (18–31)	7/31/20/0	34/11/13/0	——	8 (4–12)	25	80
Huang et al. [14]	2017	NRCT	Rob	40	60.0 ± 12.2	25/15	23.0 ± 4.4	——	——	2.5 ± 1.5	6.8 ± 3.2	——	——
			Lap	38	60.1 ± 14.2	28/10	24.3 ± 3.5	——	——	2.7 ± 1.6	6.3 ± 3.4	——	——
Park et al. [15]	2013	NRCT	Rob	40	57.3 ± 12.1	28/12	23.9 ± 2.4	27/9/4/0	1/19/19/1	2.8 ± 1.9	3.4 ± 1.1	——	6
			Lap	40	63.6 ± 10.6	25/15	24.3 ± 3.1	24/14/2/0	1/13/26/0	3.2 ± 1.9	3.6 ± 1.3	——	6
Serin et al. [16]	2015	NRCT	Rob	14	54 (41–71)	14/0	24.7 (2, 7–23, 23–27)	——	——	——	——	——	——
			Lap	65	57 (28–80)	65/0	26 (21–32)	——	——	——	——	——	——
Tang et al. [17]	2020	RCT	Rob	65	55.1 ± 12.1	36/29	22.0 ± 2.5	35/30/0/0	9/28/28/0	3.6 ± 1.1	6.0 ± 2.4	1	24
			Lap	64	58.0 ± 9.7	36/28	22.1 ± 2.3	27/37/0/0	7/32/25/0	3.7 ± 1.0	5.8 ± 2.6	0	24
Yoo et al. [18]	2014	NRCT	Rob	44	59.77 ± 12.33	35/9	24.13 ± 3.33	26/17/1/0	2/15/22/5	2.97 ± 1.75	3.24 ± 0.78	24	60
			Lap	26	60.5 ± 10.75	19/7	21.42 ± 3.13	15/11/0/0	3/4/18/1	3.62 ± 2.27	3.71 ± 0.89	7	60
Zou et al. [19]	2018	RCT	Rob	50	53.1 ± 11.8	29/21	22.0 ± 1.7	34/13/3/0	21/24/5/0	3.52 ± 1.15	5.9 ± 2.4	——	——
			Lap	50	57.8 ± 9.5	26/24	21.9 ± 1.8	30/16/4/0	23/21/6/0	3.46 ± 12.1	5.8 ± 2.7	——	——

### Risk of bias assessment

**Fig. 2:**
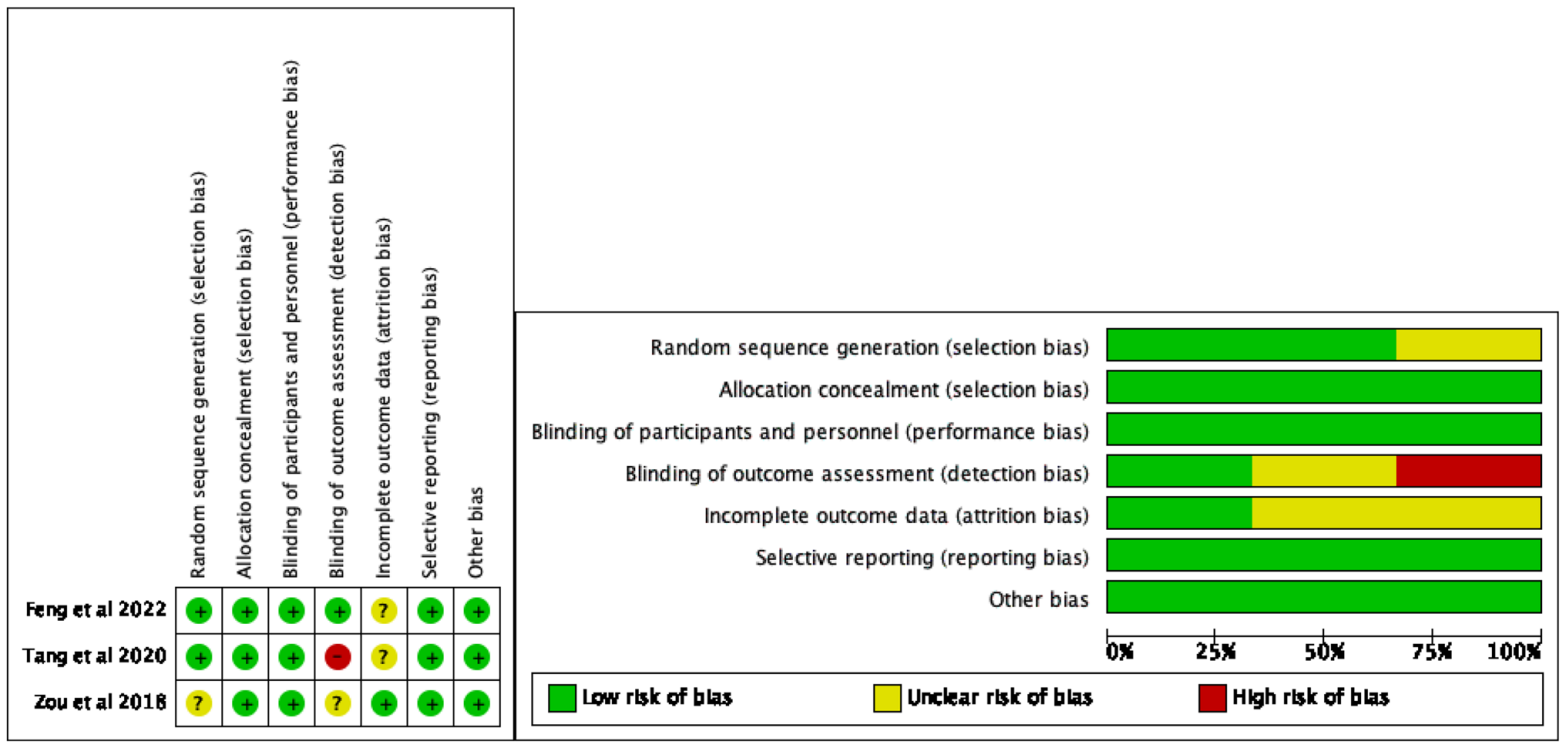
The risk of bias assessment according to the Cochrane Collaboration’s tool

**Fig. 3:**
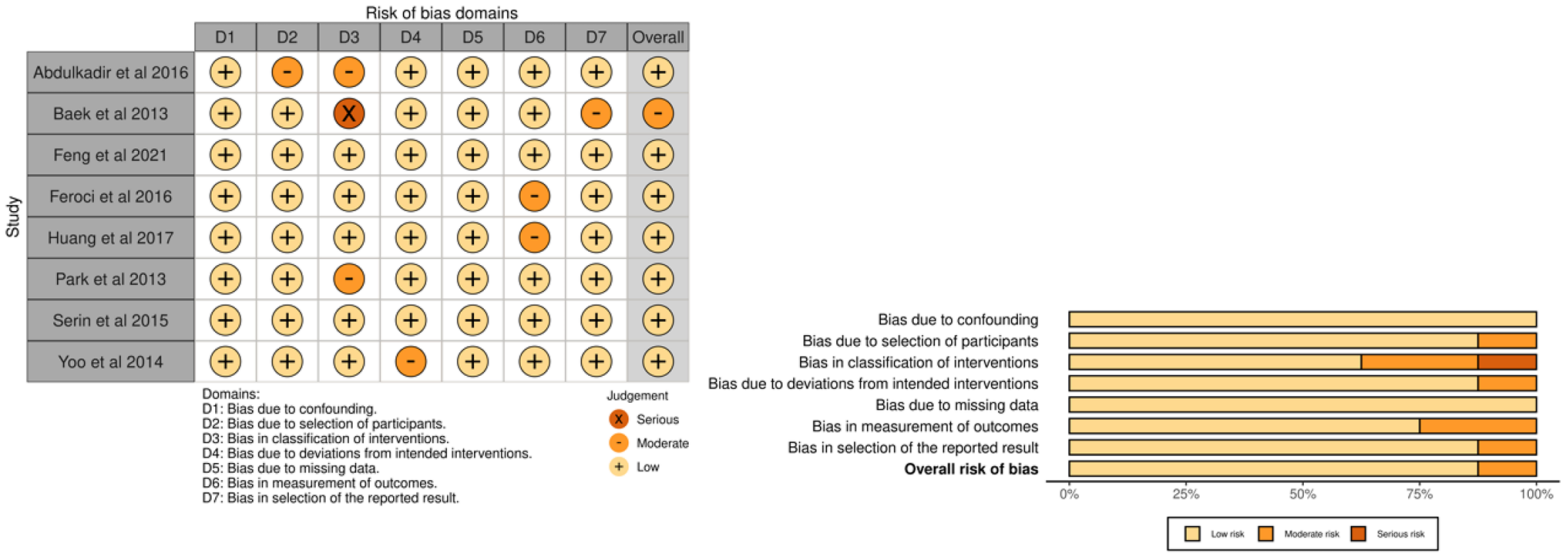
The risk of bias assessment according to the ROBINS-I tool

After assessing RCTs using the Cochrane Collaboration’s tool, it was found that one study had an unclear risk in random sequence generation, one study had a higher risk, and another study had an unclear risk in the blinding of outcome assessment. Additionally, there were two studies with an unclear risk in incomplete outcome data. The risk of bias assessment according to the Cochrane Collaboration’s tool is shown in Fig. 2. Applying the ROBINS-I tool to assess the risk of other NRCTs revealed that one study was at moderate risk, while the rest of the studies were at low risk.. The risk of bias assessment according to the ROBINS-I tool is shown in Fig. 3.

### Perioperative outcome

#### Operation time

A total of 5 study were included in the study, and there was significant heterogeneity among the included studies (*P* < 0.00001, *I*^2^ = 93%), necessitating the use of a random-effects model. The results indicated no significant difference between the two groups (MD = 11.10, 95% CI: −16.12 to 38.32, *P* = 0.42).

#### Operative blood loss

A total of 6 study were included in the study, and there was significant heterogeneity among the included studies (*P* = 0.001, *I*^2^ = 75%), necessitating the use of a random-effects model. The results indicate that the robotic group had less operative blood loss (MD =  −40.01, 95% CI: −57.6 to −22.42, *P* < 0.00001).

#### Protective stoma rate

A total of 7 study were included in the study, and there was no significant heterogeneity among the included studies (*P* = 0.10, *I*^2^ = 43%), necessitating the use of a fixed-effects model. The results indicated no significant difference between the two groups (OR = 0.95, 95% CI: 0.65 to 1.39, *P* = 0.81).

#### Conversion to open surgery rate

A total of 5 study were included in the study, and there was no significant heterogeneity among the included studies (*P* = 0.18, *I*^2^ = 38%), necessitating the use of a fixed-effects model. The robotic group had a lower rate of conversion to open surgery (OR = 0.27, 95% CI: 0.09 to 0.82, *P* = 0.02).

#### Time to flatus

A total of 5 study were included in the study and there was significant heterogeneity among the included studies (*P* = 0.004, *I*^2^ = 71%), necessitating the use of a random-effects model. The results indicated no significant difference between the two groups (SMD =  −0.20, 95% CI: −0.49 to −0.09, *P* = 0.18).

#### Time to liquid diet

A total of 5 study specify this outcomes, and there was significant heterogeneity among the included studies (*P* = 0.0001, *I*^2^ = 82%), necessitating the use of a random-effects model. The results indicated no significant difference between the two groups (SMD =  −0.11, 95% CI: −0.57 to 0.36, *P* = 0.65).

#### Total hospital stay

A total of 6 study were included in the study, and there was significant heterogeneity among the included studies (*P* = 0.04, *I*^2^ = 56%), necessitating the use of a random-effects model. The results indicated no significant difference between the two groups (MD =  −0.30, 95% CI: −0.92 to 0.32, *P* = 0.35).

#### Postoperative morbidity rate

A total of 10 study were included in the study, and there was no significant heterogeneity among the included studies (*P* = 0.72, *I*^2^ = 0%), necessitating the use of a fixed-effects model. In terms of postoperative morbidity rate, the robotic group had a lower occurrence (OR = 0.66, 95% CI: 0.53 to 0.82, *P* < 0.0001). Subgroup analysis for postoperative morbidity revealed a lower anastomotic leakage rate in the robotic group (OR = 0.66, 95% CI: 0.45 to 0.97, *P* = 0.04), with no significant differences in abdominal or anastomotic bleeding (OR = 0.65, 95% CI: 0.32 to 1.31, *P* = 0.23), wound-related complications (OR = 0.66, 95% CI: 0.39 to 1.11, *P* = 0.12), ileus (OR = 0.54, 95% CI: 0.26 to 1.13, *P* = 0.10), urinary retention or infection (OR = 0.58, 95% CI: 0.32 to 1.05, *P* = 0.07), stoma-related complications (OR = 0.86, 95% CI: 0.40 to 1.85, *P* = 0.70), and pulmonary infection (OR = 0.60, 95% CI: 0.19 to 1.85, *P* = 0.37).

#### Clavien–Dindo grade

A total of 7 study were included in the study, and there was no significant heterogeneity among the included studies (*P* = 0.90, *I*^2^ = 0%), necessitating the use of a fixed-effects model. The results indicated the robotic group’s occurrence rate of complications with Clavien–Dindo grade ≥ 3 was lower (OR = 0.60, 95% CI: 0.39 to 0.90, *P* = 0.02).

### Oncological outcomes

#### Harvested lymph nodes

A total of 4 study were included in the study, and there was no significant heterogeneity among the included studies (*P* = 0.54, *I*^2^ = 0%), necessitating the use of a fixed-effects model. The results indicated the robotic group had a higher number of harvested lymph nodes (MD = 1.97, 95% CI: 0.77 to 3.18, *P* = 0.001).

#### Proximal resection margin

A total of 4 study were included in the study, and there was no significant heterogeneity among the included studies (*P* = 0.45, *I*^2^ = 0%), necessitating the use of a random-effects model. The results indicated no significant difference between the two groups (MD =  −1.11, 95% CI: −2.54 to 0.33, *P* = 0.13).

#### Distal resection margin

A total of 5 study were included in the study, and there was no significant heterogeneity among the included studies (*P* = 0.55, *I*^2^ = 0%), necessitating the use of a random-effects model. The results indicated no significant difference between the two groups (MD = 0.09, 95% CI: −0.008 to 0.26, *P* = 0.30).

#### Circumferential resection margin positive rate

A total of 7 study were included in the study, and there was no significant heterogeneity among the included studies (*P* = 0.91, *I*^2^ = 0%), necessitating the use of a fixed-effects model. The robotic group had a lower circumferential resection margin positive rate (OR = 0.46, 95% CI: 0.23 to 0.95, *P* = 0.04).

### Long-term outcome

#### 3-year overall survival rate

A total of 3 study were included in the study, and there was no significant heterogeneity among the included studies (*P* = 0.49, *I*^2^ = 0%), necessitating the use of a fixed-effects model. The results indicated no significant difference between the two groups (MD = 1.15, 95% CI: 0.60 to 2.22, *P* = 0.67).

#### 3-year disease-free survival rate

A total of 3 study were included in the study, and there was no significant heterogeneity among the included studies (*P* = 0.90, *I*^2^ = 0%), necessitating the use of a fixed-effects model. The results indicated no significant difference between the two groups (MD = 1.23, 95% CI: 0.74 to 2.03, *P* = 0.42).

#### Publication bias

**Fig. 4:**
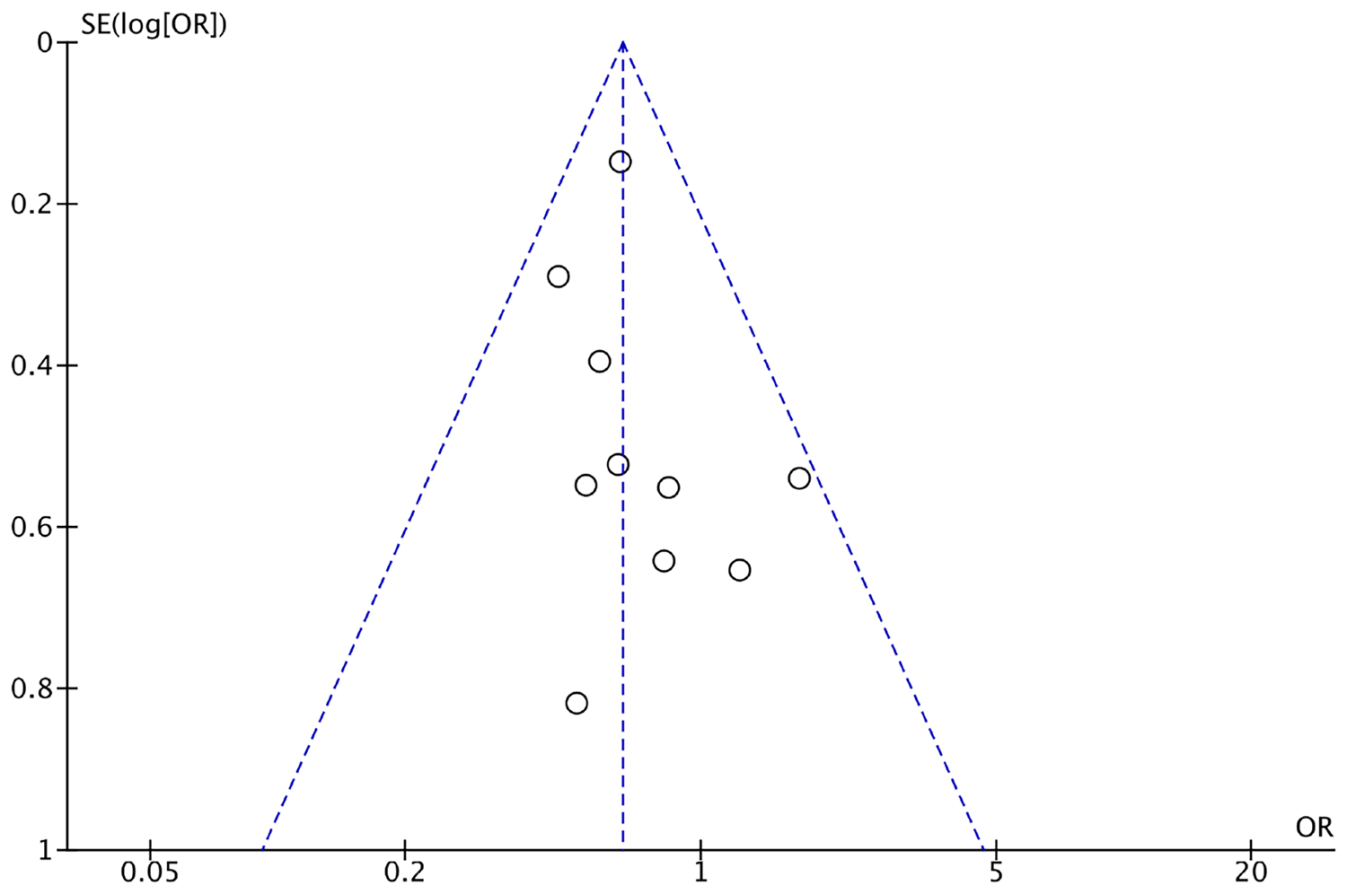
A funnel plot

A funnel plot was generated using the postoperative overall complication rates for both groups as indicators. The results showed that the scatter points representing each included study were mostly within the funnel, and the majority of scatter points were symmetrically distributed along the central axis. This suggests a low risk of bias in the included studies (Fig. 4).

### Trial sequential analysis (TSA)

**Fig. 5:**
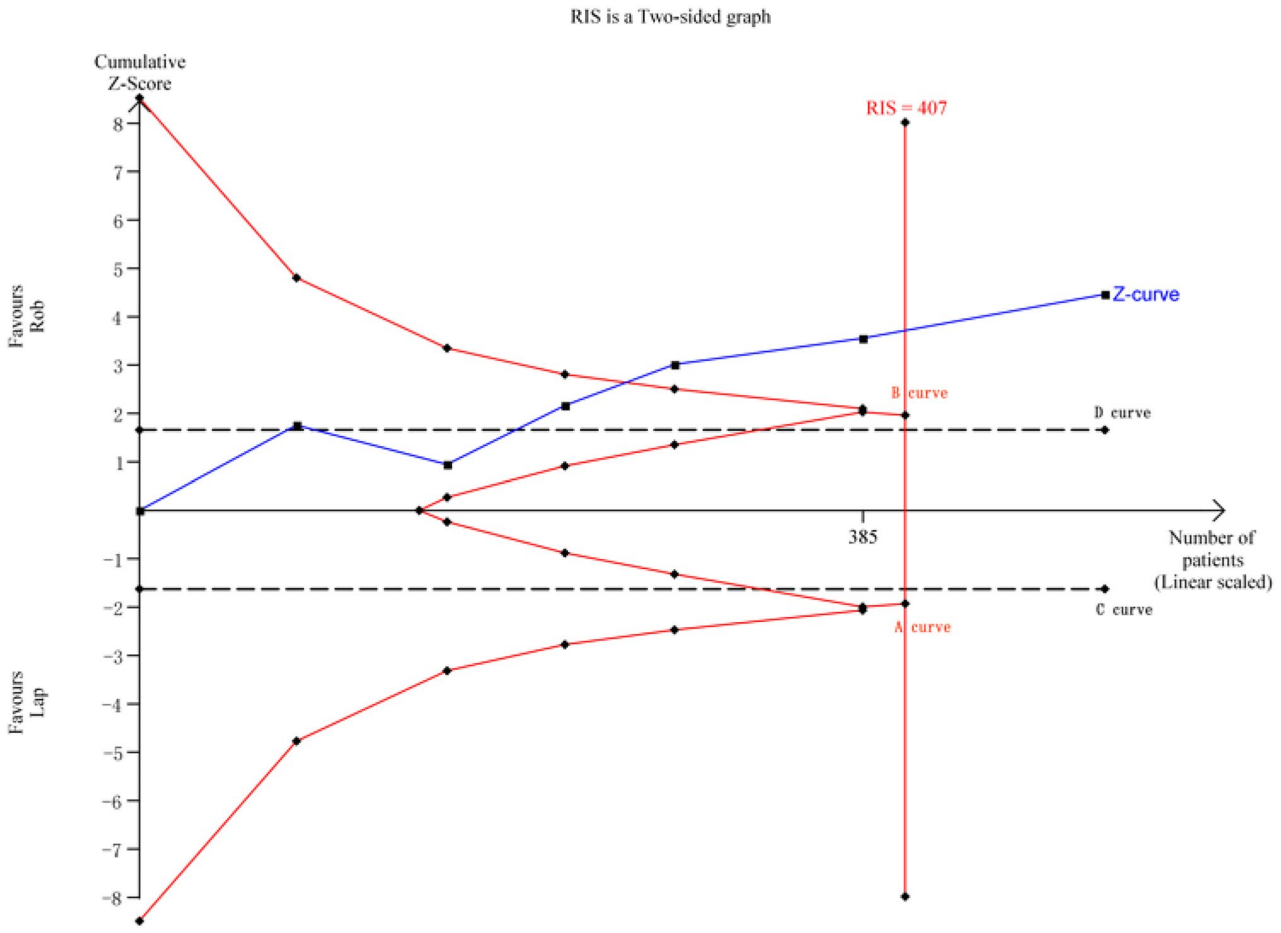
TSA for operative blood loss

For operative blood loss, a meta-analysis of 6 studies with 514 cases was conducted. The required information size for the actual meta-analysis (RIS) was 407, estimated based on the following statistical indicators: type I error rate (*α* = 0.05) and type II error rate (*β* = 0.2). TSA results demonstrated that the cumulative *Z*-value (*Z*-curve) crossed both the conventional boundary (*D*-curve) and the TSA boundary (*B*-curve), providing evidence of the superiority of the robotic group, with the cumulative information size reaching the required level (Fig. 5).

**Fig. 6:**
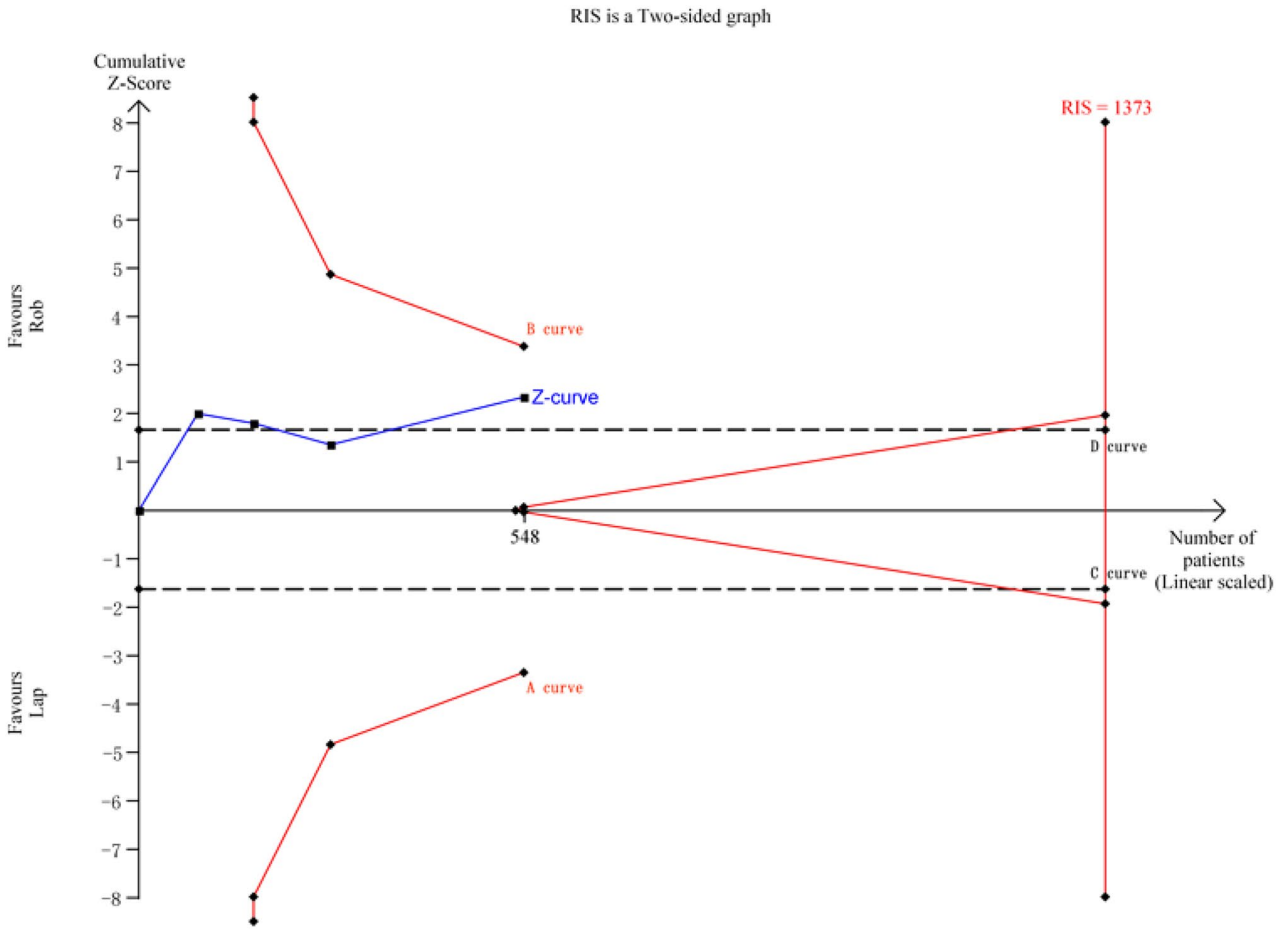
TSA for conversion to open surgery rate

For conversion to open surgery rate, a meta-analysis of 5 studies with 628 cases was performed. The RIS for the actual meta-analysis was 1373, estimated based on the specified statistical indicators. TSA results indicated that the cumulative *Z*-value (*Z*-curve) crossed the conventional boundary (*D*-curve) but did not surpass the TSA boundaries (*A*-curve and *B*-curve), suggesting a higher possibility of false positives. Further randomized controlled trials are needed to validate this outcome (Fig. 6).

**Fig. 7:**
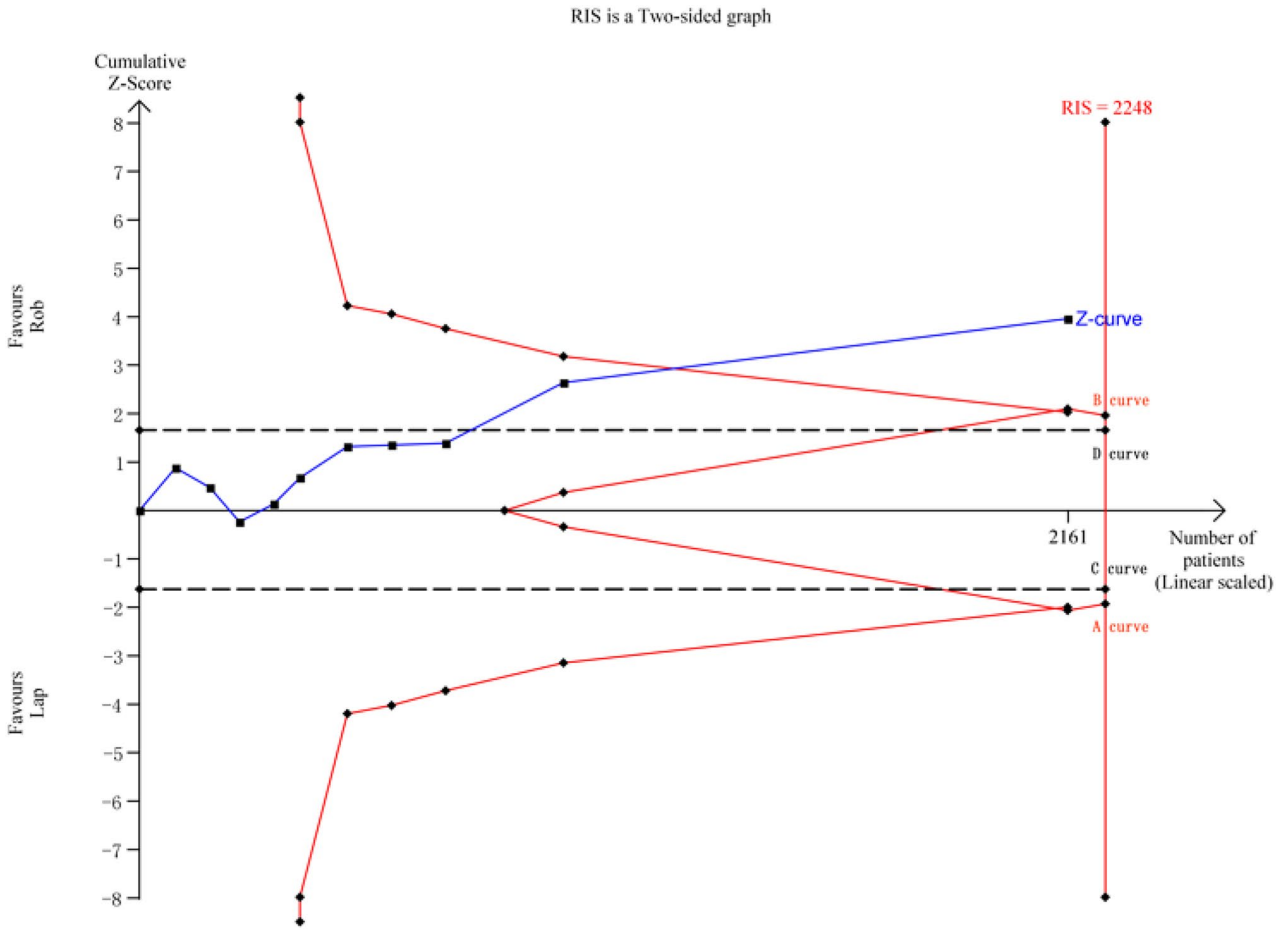
TSA for postoperative morbidity

In the case of postoperative morbidity, a meta-analysis of 10 studies with 2161 cases was conducted. The RIS for the actual meta-analysis was 2248, estimated based on the predefined statistical indicators. TSA results demonstrated that the cumulative *Z*-value (*Z*-curve) crossed both the conventional boundary (*D*-curve) and the TSA boundary (*B*-curve), providing evidence of the superiority of the robotic group. However, the cumulative information size did not reach the required level (Fig. 7).

**Fig. 8:**
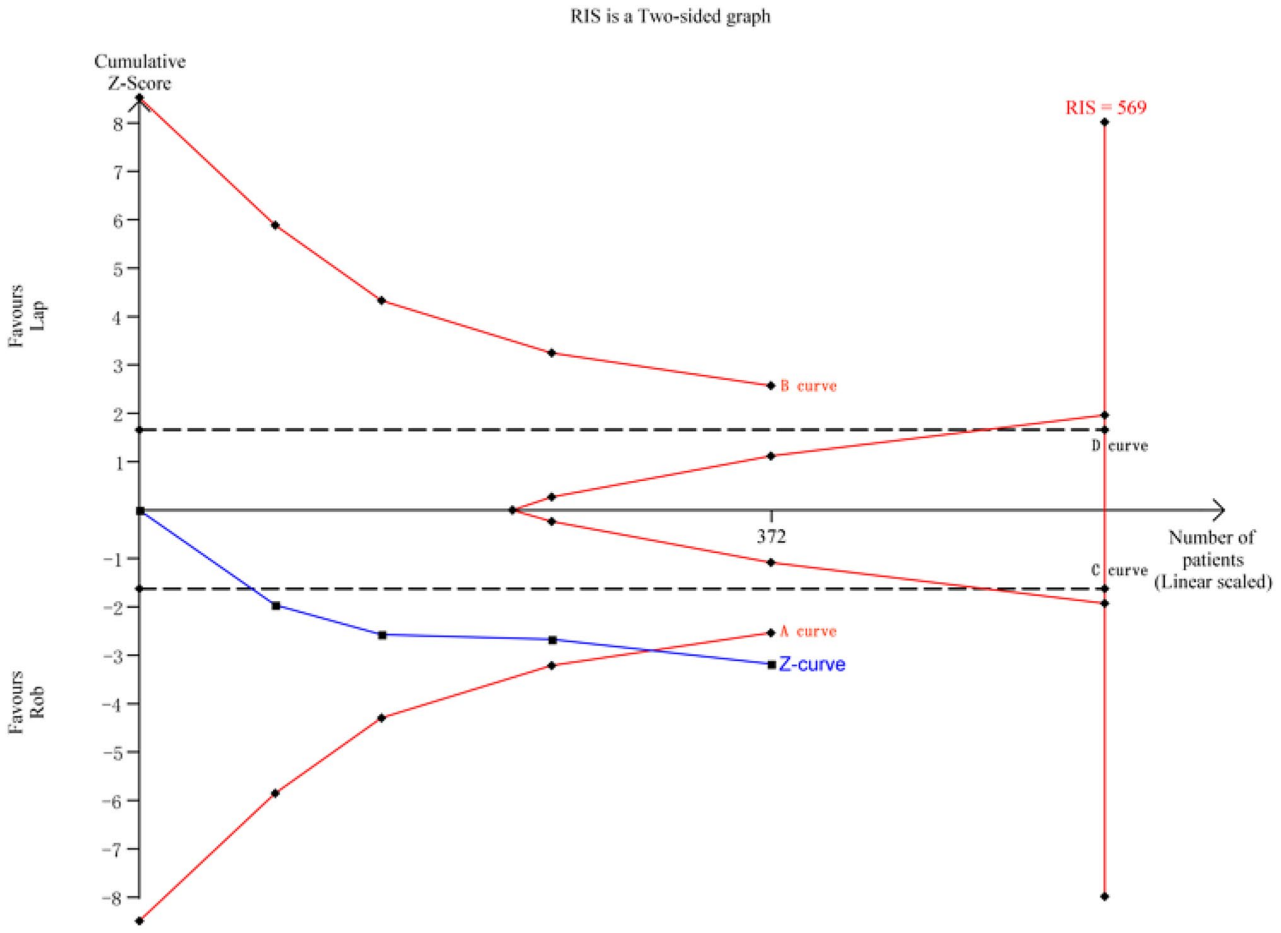
TSA for harvested lymph nodes

For harvested lymph nodes, a meta-analysis of 4 studies with 372 cases was performed. The RIS for the actual meta-analysis was 569, estimated based on the specified statistical indicators. TSA results indicated that the cumulative *Z*-value (*Z*-curve) crossed the conventional boundary (*C*-curve) and the TSA boundary (*A*-curve), providing evidence of the superiority of the robotic group. However, the cumulative information size did not reach the required level (Fig. 8).

**Fig. 9:**
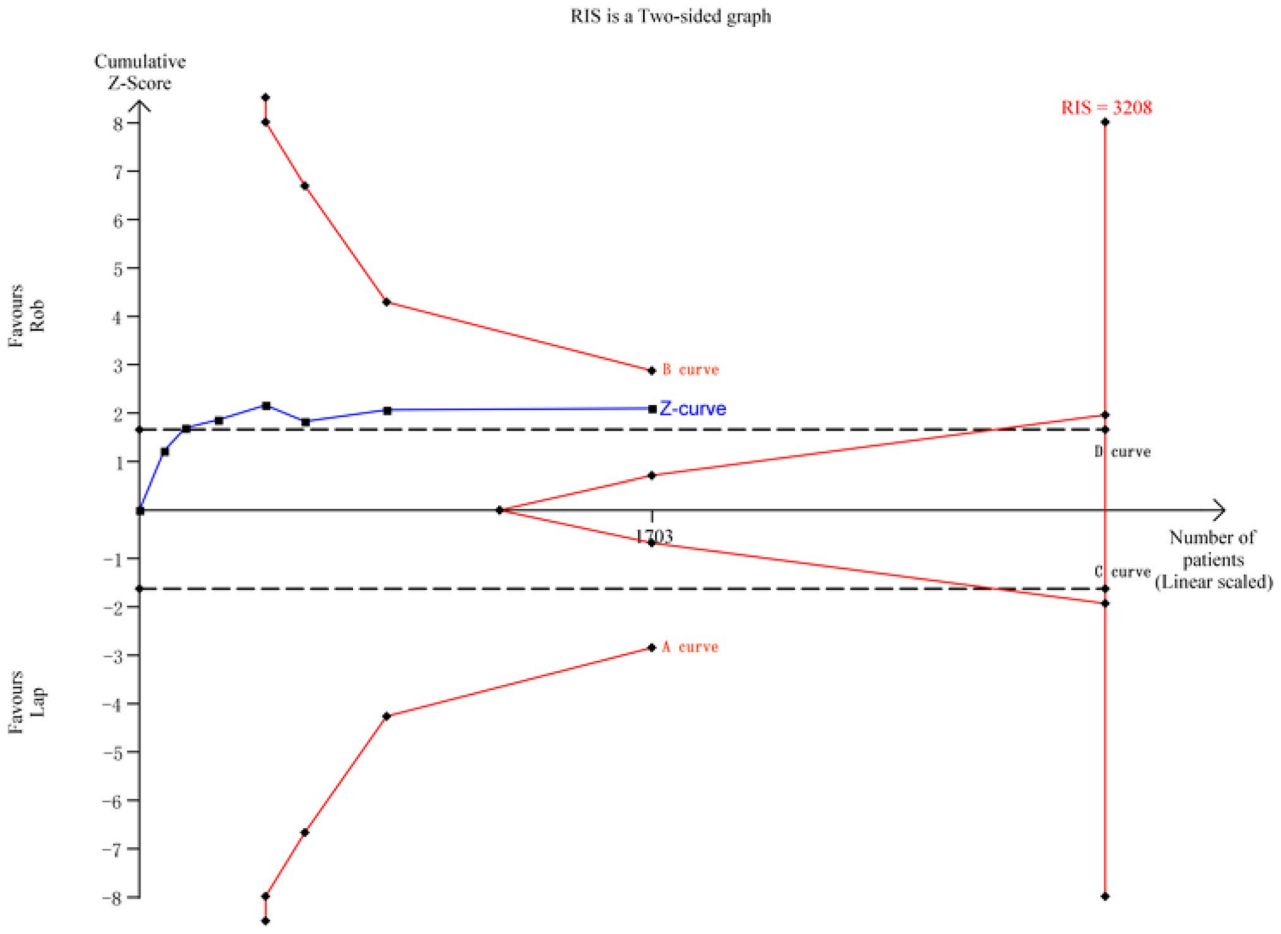
TSA for circumferential resection margin positive rate

Regarding circumferential resection margin positive rate, a meta-analysis of 7 studies with 1703 cases was conducted. The RIS for the actual meta-analysis was not specified in the provided text. TSA results showed that the cumulative *Z*-value (*Z*-curve) crossed the conventional boundary (*D*-curve) but did not surpass the TSA boundaries (*A*-curve and *B*-curve), indicating a higher possibility of false positives. Additional randomized controlled trials are required to further validate this outcome (Fig. 9).

## Discussion

Colorectal cancer (CRC) is one of the most prevalent malignant tumors globally, and its incidence is increasingly shifting toward a younger demographic [20, 21]. However, in contrast to colon cancer, surgery for middle and lower rectal cancer is more intricate, characterized by specific surgical requirements and operating environments [17, 22–24].

Total mesorectal excision (TME) is the gold standard for rectal cancer surgery, ensuring complete removal of the rectum and lymph nodes for a negative circumferential surgical margin [25]. Modern surgery prioritizes preserving pelvic nerve function, demanding exceptional skills due to the complex pelvic structure [26–29].

Entering the twenty-first century, minimally invasive surgery has become the main theme and inevitable trend in the field of colorectal surgery. Conventional laparoscopic systems have inherent drawbacks such as non-stereoscopic imaging technology, a steep learning curve, the “chopstick effect” leading to a phenomenon known as “clashing,” potential hand tremors, and the passive fatigue-inducing standing posture of the surgeon [30, 31]. These limitations have paved the way for the rise of robotic surgical systems, with the da Vinci Surgical System (DVSS) representing surgical robots that have gained global popularity due to their novel concepts and advanced technological advantages, overcoming the inherent deficiencies of laparoscopic technology [14, 32].

Controversies surround robot use in middle and lower rectal cancer treatment. Bulky robot size limits use to large operating rooms, posing challenges in surgeries on slender patients or involving multiple sites [33]. Lack of tactile feedback hinders surgeons’ sense of touch, risking tissue damage. Reliance on visual cues without tension control increases surgery complexity. Lengthy DVSS setup extends surgical time, reducing operating room efficiency. Wireless interference during surgery prolongs operation times [34, 35]. Long-term effects of robot-assisted therapy for rectal cancer are unclear. Our recent meta-analysis compared short-term and long-term outcomes of robot-assisted and laparoscopic surgery [36].

In analyzing the results, we found that robotic surgery does not increase the surgical time, achieving comparable postoperative recovery outcomes to pure laparoscopic surgery. Additionally, robotic surgery results in less intraoperative bleeding and a lower conversion rate to open surgery. On both proximal and distal resection margins, the robotic approach demonstrates similar effectiveness to laparoscopic surgery. Moreover, the robotic group exhibits a higher lymph node clearance, a lower circumferential resection margin positive rate, indicating that robotic rectal cancer surgery has better curative effects compared to laparoscopic surgery. The analysis also reveals that robotic surgery can achieve a 3-year overall survival rate and 3-year disease-free survival rate similar to laparoscopic surgery.

The robotic surgery system’s superiority lies in its simulated multi-degree-of-freedom mechanical arms, replicating human wrist articulation. The end effector’s unrestricted rotation in *Rx.Ry.Rz* directions enhances operational flexibility [37–39]. Automatic filtering of surgeon hand tremors and a motion calibration system ensure instrument stability, reinforcing surgical operation stability. In confined spaces, the system enables precise tasks like dissection, hemostasis, and suturing. A stable 10 × high-definition magnification offers a three-dimensional visual field, improving hand–eye coordination [40]. Ergonomic design follows human engineering principles, reducing fatigue, maintaining focus, and lowering error rates during complex procedures [41].

Overall, postoperative complications are crucial indicators for assessing the safety and feasibility of surgical procedures. Therefore, we further discussed the advantages and disadvantages of robotic and laparoscopic surgeries from the perspective of postoperative complications. The meta-analysis results indicate a lower incidence of postoperative complications and a lower rate of complications with Clavien–Dindo grade ≥ 3 for robotic surgery, consistent with findings from multiple studies. Consequently, we conclude that, compared to laparoscopic surgery, robotic rectal cancer surgery holds distinct advantages. Further subgroup analysis reveals that the robotic group has a lower rate of anastomotic leakage, a critical complication after radical rectal resection. Anastomotic leakage-induced acute diffuse peritonitis is the most severe complication following rectal surgery, leading to reoperation or even death [42]. Anastomotic leakage, usually caused by low anastomotic position, poor blood flow, tension, and local infection [43], occurs at a rate of 5.6% in the robotic group and 8.3% in the laparoscopic group in this meta-analysis. DVSS’s low anastomotic leakage is due to precise robotic arms, advanced imaging, and stability, ensuring accurate and stable anastomosis during the procedure.

Moreover, it is noteworthy that urinary complications are one of the parameters used to assess the protection of pelvic autonomous nerves during surgery. Although urinary complications are considered to be caused by various factors, surgical injury during the procedure is considered a major contributor, significantly impacting postoperative quality of life. Previous studies [44–47] suggested that robotic rectal surgery can significantly protect pelvic autonomous nerves due to its 10-fold magnification of the surgical field, leading to a substantial reduction in the incidence of postoperative urinary complications. However, in our meta-analysis, no statistically significant difference was observed between robotic and laparoscopic rectal surgeries regarding urinary complications.

This study has some limitations that should be noted. Firstly, the relatively small sample size included in the literature may limit a comprehensive evaluation of robotic treatment for low rectal cancer. Due to the limited sample size, the observation period of the study is relatively short, providing limited insight into long-term treatment outcomes. Long-term follow-up is crucial for evaluating the sustained effects of surgery and factors such as patient survival. Therefore, future research should focus on expanding the sample size and adopting a more extended observation period to obtain more comprehensive and reliable data.

Secondly, the lack of a large number of high-quality RCTs is also a limitation of the study. RCTs are generally considered the gold standard for assessing the effectiveness of treatment methods. Still, in this study, relevant RCT studies were relatively limited. This may introduce potential bias and uncertainty, affecting the accurate assessment of the effectiveness of robotic treatment. To draw more confident and reliable conclusions about the effectiveness of robotic treatment for low rectal cancer, future research needs more support from high-quality RCT study.

Given these limitations, we hope that future studies can overcome these challenges by expanding the sample size, extending the observation period, and increasing the number of RCT studies, providing more reliable and comprehensive clinical evidence. This will contribute to a deeper understanding of the actual effects of robotic treatment for low rectal cancer and offer more scientific and reliable guidance for clinical practice.

## Conclusion

Robot-assisted laparoscopic treatment for mid and low rectal cancer yields favorable outcomes, demonstrating both efficacy and safety. In comparison to conventional laparoscopy, patients experience reduced intraoperative bleeding and a lower incidence of complications. Notably, the approach achieves comparable short-term and long-term treatment results to those of conventional laparoscopic surgery, thus justifying its consideration for widespread clinical application.

## Supplementary Information

Below is the link to the electronic supplementary material.

Supplementary file1 (DOCX 414 KB)

## Data Availability

Data sharing was not applicable to this article, as no datasets were generated or analyzed during the current study.
