# Supplementary material for: Short-term and long-term efficacy in robot-assisted treatment for mid and low rectal cancer: a systematic review and meta-analysis
Source: Int J Colorectal Dis. 2023 Dec 21;39(1):7. doi: 10.1007/s00384-023-04579-3 (PMC10739549; doi:10.1007/s00384-023-04579-3)
Supplement: Supplementary file 1 — Supplementary file1 (DOCX 414 KB) [file 384_2023_4579_MOESM1_ESM.docx]

A forest plot and Subgroup analysis comparing the Operation time between the two groups

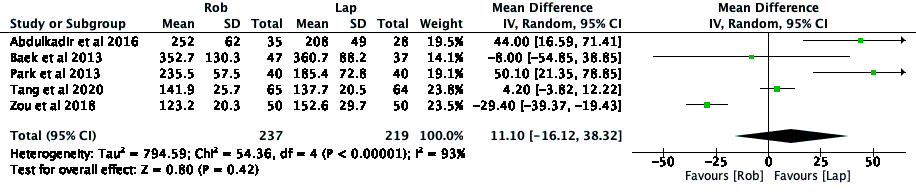


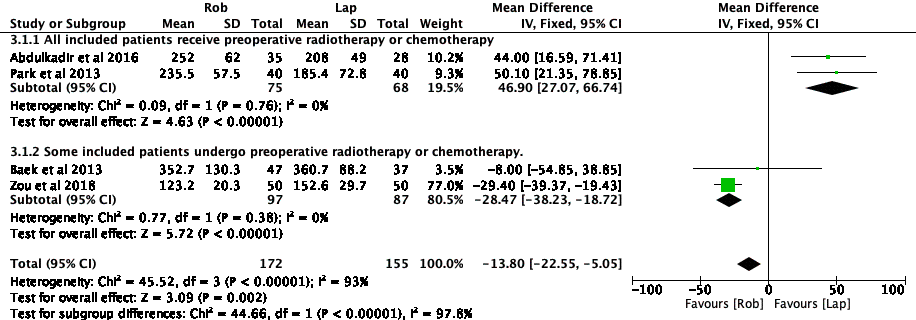

A forest plot and Subgroup analysis comparing the operative blood loss between the two groups
**
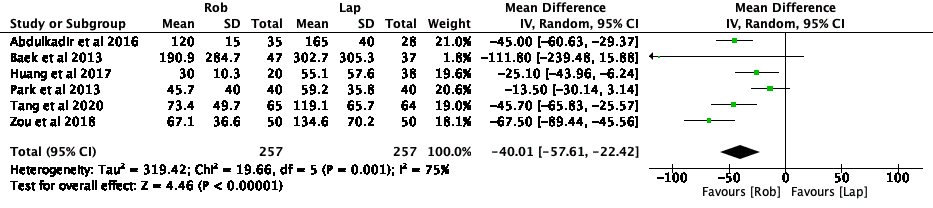
**

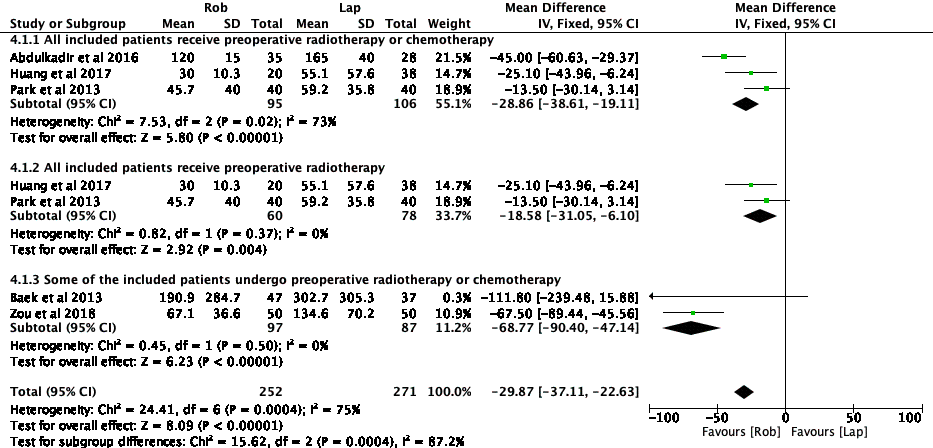

A forest plot comparing the protective stoma rate between the two groups
**
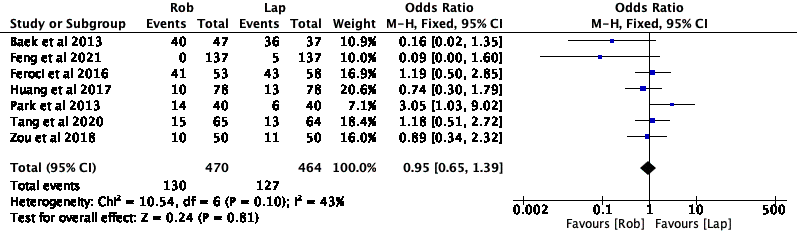
**
A forest plot comparing the conversion to open surgery rate between the two groups

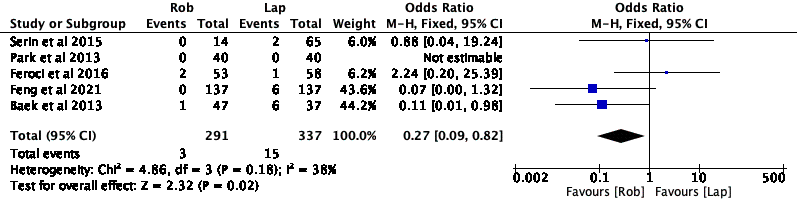

A forest plot and Subgroup analysis comparing the time to flatus between the two groups


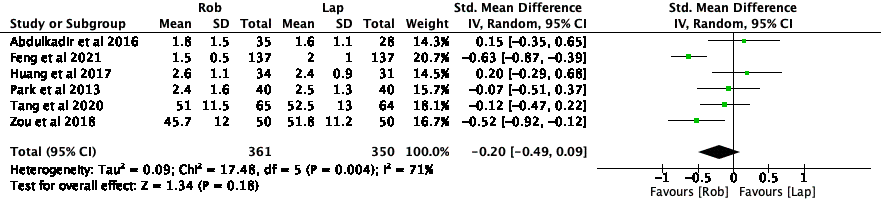


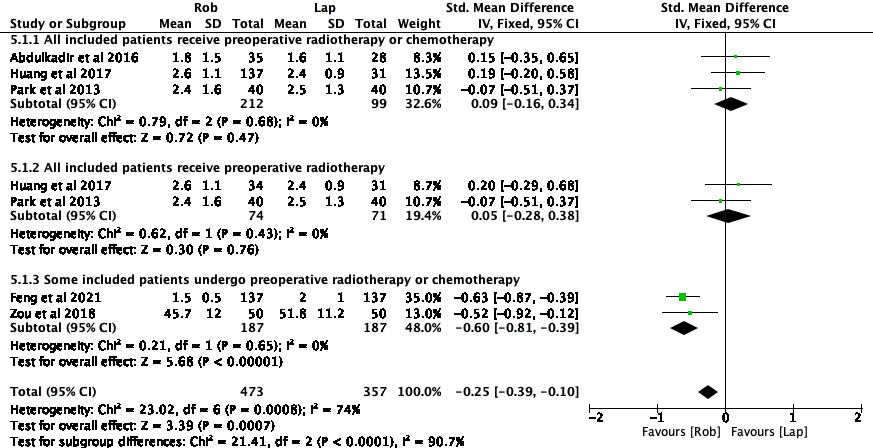

A forest plot and Subgroup analysis comparing the time to liquid diet between the two groups

**
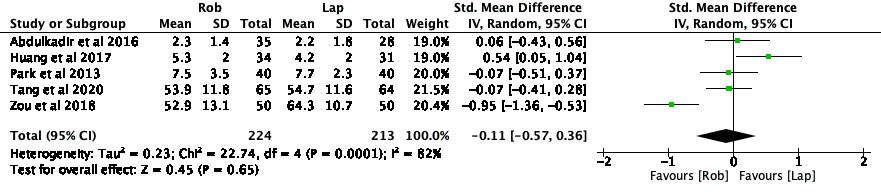
**

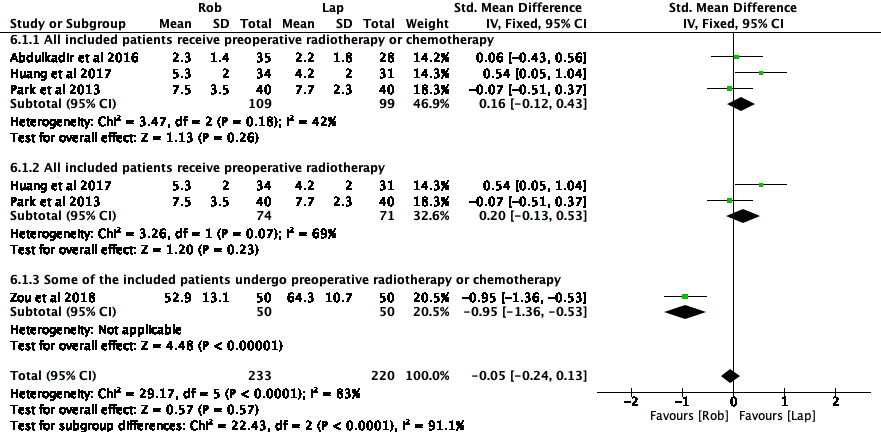

A forest plot and Subgroup analysis comparing the total hospital stay between the two groups

**
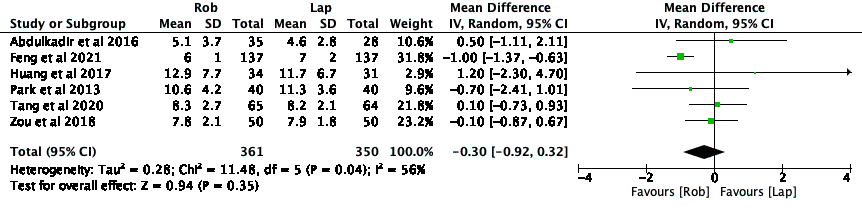
**
A forest plot comparing the postoperative morbidity rate between the two groups

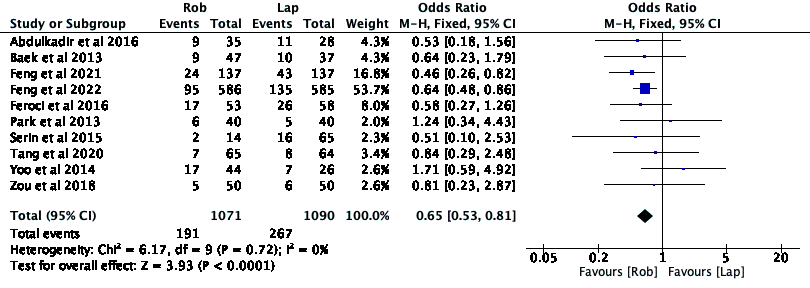

Subgroup analysis for postoperative morbidity

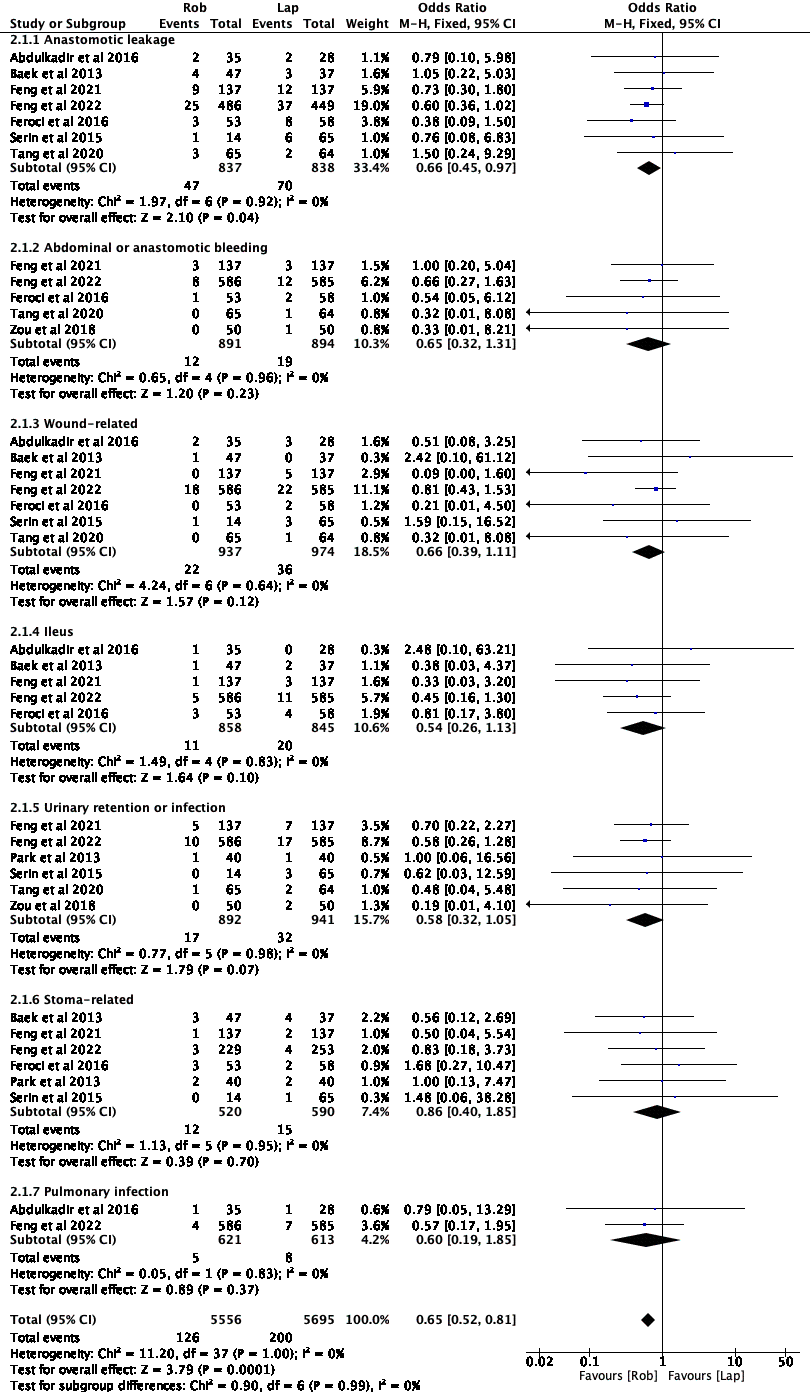

A forest plot comparing the occurrence rate of complications with Clavien–Dindo grade ≥3 between the two groups
**
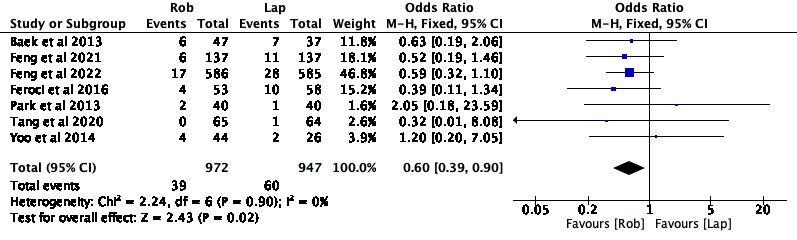
**
A forest plot comparing the harvested lymph nodes between the two groups

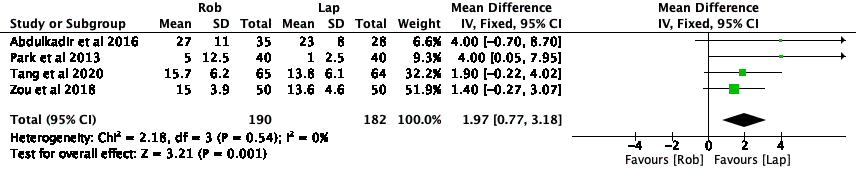

A forest plot comparing the proximal resection margin between the two groups

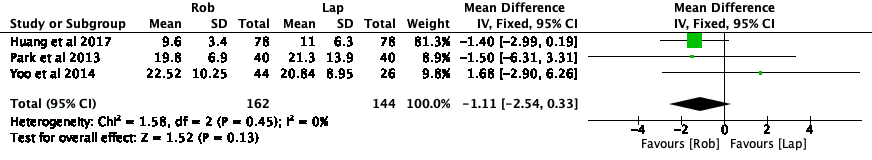

A forest plot comparing the distal resection margin between the two groups


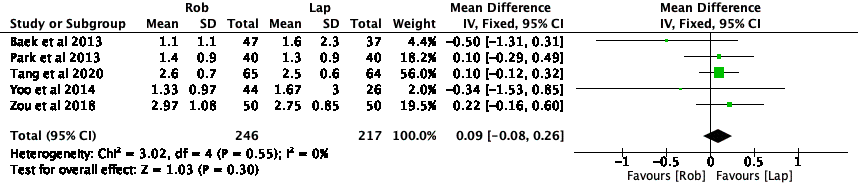

A forest plot comparing the circumferential resection margin positive rate between the two groups

**
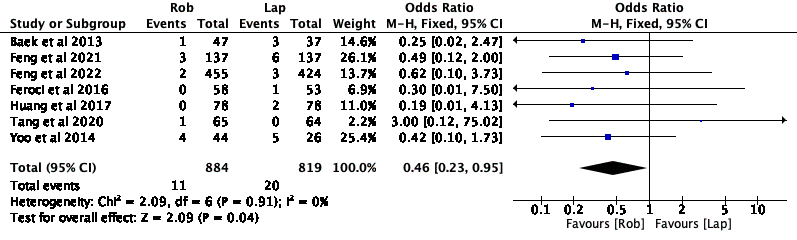
**
A forest plot comparing the 3-year overall survival rate between the two groups
**
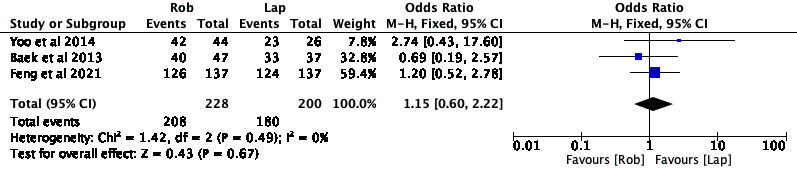
**
A forest plot comparing the 3-year disease-free survival rate between the two groups
**
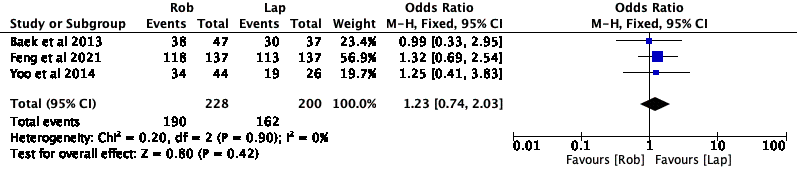
**
